# Current State, Needs, and Opportunities for Wearable Robots in Military Medical Rehabilitation and Force Protection

**DOI:** 10.3390/act13070236

**Published:** 2024-06-24

**Authors:** Rory A. Cooper, George Smolinski, Jorge L. Candiotti, Shantanu Satpute, Garrett G. Grindle, Tawnee L. Sparling, Michelle J. Nordstrom, Xiaoning Yuan, Allison Symsack, Chang Dae Lee, Nicola Vitiello, Steven Knezevic, Thomas G. Sugar, Urs Schneider, Verena Kopp, Mirjam Holl, Ignacio Gaunaurd, Robert Gailey, Paolo Bonato, Ron Poropatich, David J. Adet, Francesco Clemente, James Abbas, Paul F. Pasquina

**Affiliations:** 1 Human Engineering Research Laboratories, VA Pittsburgh Healthcare System and University of Pittsburgh, Pittsburgh, PA 15026, USA; 2 Department of Physical Medicine and Rehabilitation, Uniformed Services University of the Health Sciences, Bethesda, MD 20814, USA; 3 Department of Occupational Therapy, Indiana University Indianapolis, Indianapolis, IN 46202, USA; 4 BioRobotics Institute, Scuola Superiore Sant’Anna, 56025 Pontedera, PI, Italy; 5 Spinal Cord Damage Research Center, James J. Peters VA Medical Center, Bronx, NY 10468, USA; 6 Barrett, The Honors College, ASU Polytechnic, Mesa, AZ 85281, USA; 7 Fraunhofer Institute for Manufacturing Engineering and Automation IPA, 70569 Stuttgart, Germany; 8 Department of Physical Therapy, University of Miami Miller School of Medicine, Coral Gables, FL 33146, USA; 9 Bruce W. Carter VA Medical Center, Miami, FL 33125, USA; 10 Harvard School of Medicine, Boston, MA 02115, USA; 11 Center for Military Medicine Research, University of Pittsburgh, Pittsburgh, PA 15219, USA; 12 U.S. Army Combat Capabilities Development Command Soldier Center, Natick, MA 01760, USA; 13 Prensilia S.r.l., 56025 Pontedera, PI, Italy; 14 Institute for Integrative and Innovative Research (I3R) and the Department of Biomedical Engineering, University of Arkansas, Fayetteville, AR 72701, USA

**Keywords:** military workforce, musculoskeletal injuries, injury prevention, exoskeleton, disability, delphi method

## Abstract

Despite advances in wearable robots across various fields, there is no consensus definition or design framework for the application of this technology in rehabilitation or musculoskeletal (MSK) injury prevention. This paper aims to define wearable robots and explore their applications and challenges for military rehabilitation and force protection for MSK injury prevention. We conducted a modified Delphi method, including a steering group and 14 panelists with 10+ years of expertise in wearable robots. Panelists presented current wearable robots currently in use or in development for rehabilitation or assistance use in the military workforce and healthcare. The steering group and panelists met to obtain a consensus on the wearable robot definition applicable for rehabilitation or primary injury prevention. Panelists unanimously agreed that wearable robots can be grouped into three main applications, as follows: (1) primary and secondary MSK injury prevention, (2) enhancement of military activities and tasks, and (3) rehabilitation and reintegration. Each application was presented within the context of its target population and state-of-the-art technology currently in use or under development. Capturing expert opinions, this study defines wearable robots for military rehabilitation and MSK injury prevention, identifies health outcomes and assessment tools, and outlines design requirements for future advancements.

## 1. Introduction

The human body is susceptible to sustaining musculoskeletal (MSK) injuries due to repetitive movements or limb overuse. These injuries are a common problem among U.S. military Service members, Veterans, and healthcare service providers during object lifting, high-speed changes of direction while wearing heavy personal equipment, and patient repositioning. Over 2 million clinical visits related to MSK were reported across military services in 2017, resulting in 8 million limited-duty days [1]. The incidence of MSK problems among military personnel surpasses that of the general population by more than tenfold [2], being the number one reason for the Department of Defense’s medical issue that limits force readiness [2,3].

Similarly, in 2020, the Healthcare and Social Assistance sector (HCSA) had over 806,200 private industry injury and illness cases [4]. Among these cases, nursing assistants, registered nurses, and licensed practical and vocational nurses—who are frequently involved in manual patient handling, including lifting, moving, and repositioning—experienced notable increases in the number of days away from work [4]. The effects of MSK injuries can lead to chronic disability [5], delays in military readiness [6], and high medical expenses [7].

Wearable robots represent a novel technological advancement for averting work-related injuries [8]. Wearable robots are devices attached to the human body to enhance or assist motor functions. The technology may empower individuals to execute tasks with reduced physical strain, enhanced efficiency, and prolonged task and mission endurance [9,10]. Additionally, wearable robots may contribute to the recovery process for those with injuries and disabilities, facilitating their rehabilitation and successful reintegration into society [11–13]. Despite the rapid development of wearable robot technology in various fields, there is no consensus on the definition and framework of wearable robots for military rehabilitation and assistance in preventing MSK injuries. Proud et al. [14] highlight that evaluation procedures for exoskeletons vary widely across different fields, complicating comparisons. They also emphasize that significant adaptation of existing technologies, such as industrial exoskeletons, would be necessary to meet the specific requirements of military applications. Meanwhile, other studies focus on the design considerations for lower [15] and/or upper exoskeletons [15] and/or upper exoskeletons [16] tailored for military use, but they often neglect alternative wearable robots designed particularly for rehabilitation and reintegration purposes. Therefore, this paper aims to define wearable robots within this context and to describe categories of wearable robots employed in the prevention of MSK injuries in the support of military operations and in the enhancement of rehabilitation outcomes and social reintegration efforts. Our findings assess the current state of technology, design requirements and challenges, and research and development recommendations.

## 2. Materials and Methods

We used a modified Delphi process guided by a steering group to establish consensus on the wearable robot definition and categories among a panel of experts. The study was conducted at a 2-day workshop at the State of Science Symposia organized by the Center for Rehabilitation Science Research (CRSR) within the Uniformed Services University of the Health Sciences (USUHS) and the Human Engineering Research Laboratories (HERL) of the University of Pittsburgh and US Department of Veterans Affairs, who served as the steering group. The Delphi method is a qualitative analysis by which a group of experts share their opinions to develop best-practice guidance where research is limited or evidence is conflicting [17]. Its modified version is a formal panel consensus process guided by a steering group achieve a convergence of opinion among the group of experts. Studies have demonstrated that the modified Delphi method can be superior to the original Delphi method, and is perceived to be highly cooperative and effective [18,19]. The Delphi process involves multiple rounds of controlled feedback. Conflicts were resolved by using a diverse panel of experts chosen to ensure a wide range of perspectives. The selection criteria included expertise, experience, and relevance to wearable robotics for MSK prevention, and those who had an existing or prior collaboration with the US Department of Defense or US Department of Veterans Affairs. Experts were asked to provide a presentation summarizing the relevant literature on wearable robot applications for MSK injury prevention and military medical rehabilitation, technology currently in use or under development, and design requirements and challenges, with a set of questions and statements related to wearable robotics. During round 1, common themes, agreements, and disagreements were identified by the steering group team. Experts received a summary of the group’s responses from their presentations and any comments. In Round 2, the panel reviewed this feedback and revised initial positions in light of the group’s collective input. The process of feedback, review, and response was repeated to reduce the range of answers and move towards a consensus. Experts were encouraged to justify their opinions, especially if they deviated significantly from the group’s response. Controlled feedback ensured that experts were aware of the collective viewpoints and the reasons behind differing opinions. This helped in understanding the rationale of other experts and led to convergence. Additional rounds allowed experts to refine their views and address any misunderstandings. Conflicts were resolved as experts adjusted their opinions based on new information and insights gained from the feedback. A neutral facilitator group guided the process helped clarify misunderstandings and ensured that all voices were heard. After over four rounds, a consensus was achieved. The final result was a well-rounded agreement that reflected the collective judgment of the expert panel.

On Day 1, 14 panelists specializing in wearable robots presented their work on the current state of wearable robots used in rehabilitation and military medicine. The focus was on mitigating injuries, both primary and secondary, and accelerating rehabilitation and reintegration. Generally, Delphi sample sizes aim to achieve a panel of 11–30 members to ensure effectiveness and reliability [20,21].

A theme analysis was conducted on the presented literature using a codebook that covered aspects such as the target population, wearable robots in research and development or used in functional and clinical applications, challenges, and potential applications. Transcripts from each presentation were filtered and independently reviewed by three reviewers. The results were compared for agreement. On Day 2, the steering group met in person with panel members to reach a consensus on the themes identified in the theme analysis.

## 3. Results

The steering group and panel members agreed upon the definition that a wearable robot is a powered mechanical device with built-in sensors in segments and/or joints designed around the shape and functions of the user to restore and/or augment their physical performance. It encompasses a range of devices, including exoskeletons, powered prostheses and orthoses, and robotic power wheelchairs, among others. There was unanimous agreement that advances in wearable robots in the field of military medicine and rehabilitation can be grouped into three main application fields: (1) primary and secondary MSK injury prevention, (2) enhancement of military activities and tasks, and (3) functional rehabilitation and social reintegration. Each application was presented within the context of its target population and the state-of-the-art technology in use or under development. The discussion included health outcomes, assessment tools, technological challenges, and recommendations. An overall classification of wearable robots is described in Table 1.

### 3.1. Primary and Secondary MSK Injury Prevention

Several wearable robots for primary and secondary MSK injury prevention are currently in use or under development across various sectors, including in military, healthcare, manufacturing, construction, and industrial applications [24]. Their focus is particularly on reducing loading, fatigue, joint instability, and the risk of joint-related issues. Wearable robots can assist by augmenting the user’s strength and endurance, allowing people to sustain performance for longer periods without experiencing excessive fatigue, a common issue in military personnel activities that require prolonged physical effort.

**Figure 1.**
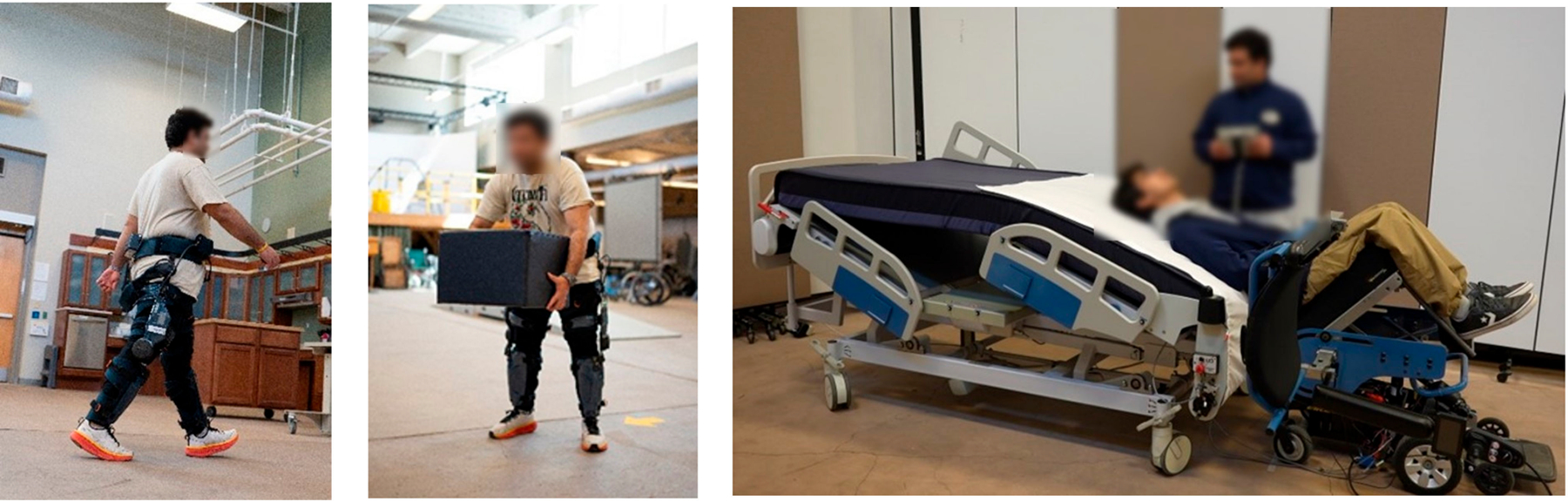
Wearable robots for primary and secondary MSK injury prevention. From left to right: The Keego exoskeleton used for walking assistance and object lifting. The Powered Personal Transfer System used by healthcare workers during transfers.

Current technologies currently in use and development include exoskeletons and assistive mobility devices. Exoskeletons are designed to enhance stability and control, particularly for individuals working in environments or with health conditions that may pose a risk for joint instability. By providing additional support to the MSK system, wearable robots can help mitigate the risk of falls or injuries associated with compromised joint stability, improving overall mobility and function. The Sarcos Guardian XO Full-Body Exoskeleton [25] is a full-body exoskeleton designed for industrial applications, including heavy lifting and manipulation, and has been assessed in military settings. The XOS 2 Exoskeleton is designed for tasks involving heavy lifting, and has been shown to reduce the physical strain on workers in manufacturing and logistics [26]. Assistive mobility devices align with the definition of a wearable robot by integrating powered mechanisms and sensor technology to enhance the mobility and function of the user. For example, the Powered Personal Transfer System (Figure 1) developed at HERL incorporates a customized power wheelchair and hospital bed to facilitate automated transfers between both systems, minimizing the risk of MSK injuries in caregivers and wheelchair users [27].

### 3.2. Enhancement of Military Activities and Tasks

**Figure 2.**
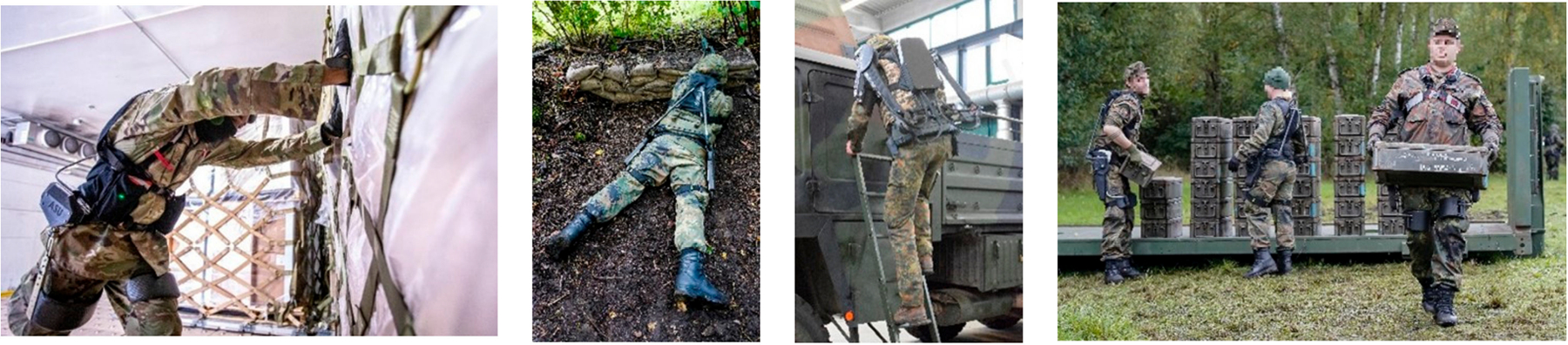
Wearable robots for occupational military activities. From left to right: Aerial Porter pushing large pallets, a soldier with exoskeleton kneeling on the ground in a defensive position, a soldier climbing a ladder with an exoskeleton, and soldiers transporting heavy boxes with an exoskeleton support them during lifting.

Soldiers undertake an inherently potentially perilous profession. Strenuous military tasks impose tremendous physical and cognitive demands on soldiers’ bodies, frequently approaching human safety limits leading to a high risk for MSK injuries [28,29]. The high frequency of repetitive actions and prolonged exposure to static stress intensifies soldiers’ fatigue, discomfort, and pain, sometimes resulting in both acute and chronic injuries. Occupational safety regulations recommend technical and organizational improvements to reduce identified MSK injury risks in civil and military work scenarios. Wearable physical assistance, in addition to improved physical training, is frequently considered after these primary approaches cannot adequately mitigate the risks (Figure 2). Exoskeletons may enhance the mission performance of military personnel by reducing physical strain on the body [30–32]. Additionally, exoskeletons may mitigate mental fatigue, thereby preserving the capacity to carry out cognitive and physical tasks effectively [33]. For instance, devices could provide support during activities, such as walking while load-bearing or while lifting and loading heavy objects (e.g., munition boxes, gas cans, etc.), as well as in the maintenance of airplanes, large vehicles, and mobile logistics.

Exoskeletons may be able to play a critical role in military missions in the following three ways: increased endurance, reduced risk of injury, and amplification of capabilities. They could affect the body mechanically by reducing the loading or by altering the load path [22,34]. In addition, the future of exoskeletons on the battlefield may evolve from the current individual limb power assistance to whole-body power assistance, perhaps ultimately evolving into a smart personal exoskeleton [16]. A smart personal exoskeleton could not only augment a soldier’s physical capabilities, but may also be integrated with combat control systems to improve the soldier’s survivability. Future directions may incorporate technologies to facilitate evacuation in case a warrior becomes incapacitated.

Wearable robots, such as the Lockheed Martin’s ONYX [16], are intended to support soldiers and workers by reducing the physical strain associated with carrying heavy loads, and has been demonstrated to be effective in both military and industrial scenarios. The ExoBoot, developed by Dephy Inc. (MA, USA), is a military-powered ankle exoskeleton that has shown positive results in reducing metabolic cost while walking [35]. Soft wearable robots are alternative exoskeletons for reducing the metabolic rate when walking and running using versatile and portable exosuits [36].

### 3.3. Functional Rehabilitation and Social Reintegration

Regaining or maintaining functional mobility is often a primary goal for individuals with impairments in neurologic and musculoskeletal function, such as limb dysfunction, spinal cord injury (SCI), and neuromuscular diseases. Wearable robots may offer new and alternative approaches for improved mobility and functionality, and may be useful in facilitating daily activity engagement by improving task performance in work environments and fostering greater social integration. For instance, individuals who have had a stroke may experience paresis and/or paralysis that limits lower and upper extremity function. Wearable robots can play a role in enhancing neuroplasticity through motor relearning for such individuals, and may contribute to improved motor control and overall rehabilitation outcomes.

A variety of exoskeletons have been developed to restore independence in mobility and activities of daily living. The MyoPro is a myoelectric orthosis that has been designed to assist individuals with upper extremity motor impairments. It detects the user’s muscle signals to power the movement of the affected arm, providing support and assistance during arm movements. Similarly, the Hybrid Assistive Limb by Cyberdyne [37] is a powered exoskeleton designed to assist and enhance human limb function via the detection of bioelectric signals from the user’s muscles to predict and support their movements.

Exoskeleton-assisted walking (EAW) devices enable users to undergo locomotion rehabilitation. Early models of exoskeletons for gait training were bulky and required assistance to use, limiting their application to clinical settings rather than personal use. However, as exoskeletons continue to become lighter, more affordable, and capable of independent use, they may become suitable for home and community use. Examples of newer devices include Ekso (Ekso Bionics, San Rafael, CA, USA), [38] Indego (Vanderbilt University, Nashville, TN, USA), [39] and ReWalk (ReWalk Robotics, Inc., Marlborough, MA, USA) [40]. EksoNR (Next-Gen Rehabilitation Exoskeleton) is designed for rehabilitation centers, and provides robotic-assisted gait training via adaptive support that may assist patients in regaining or improving their ability to walk. ReWalk offers wearable robotic exoskeletons that enable individuals with spinal cord injuries to stand, walk, and climb stairs. Rex Bionics (Rex Bionics Ltd., Auckland, New Zealand) offers a hands-free robotic exoskeleton that allows individuals with mobility impairments to stand, walk, and move in some settings in a controlled and stable manner. Other limb-specific EAW devices are intended to enhance gait patterns, including the Active Knee Orthosis (AKO) and the Active Pelvis Orthosis (APO) [41].

Recent technological advances within the field of prosthetics have also been focused on enabling individuals with limb amputations to regain or improve mobility and better participate in daily activities. Wearable applications that use inertial measurement unit (IMU) sensors systems, such as the Rehabilitation Lower-limb Orthtopedic Assistive Device (RELOAD), can assess the gait and provide audible feedback in real-time to improve walking and mobility in the home and community while reducing some of the burdens related to in-person-only physical therapy programs [42]. Myoelectric and powered prostheses have been developed to enhance functions beyond the capabilities of body-powered or mechanical components. The Power Knee (Össur, Iceland) is a motor-powered microprocessor knee with the potential to enhance gait patterns via active assistance. The Mia hand [43] is a myoelectric prosthetic hand combined with implanted magnets to provide biofeedback and a myoneural interface for more intuitive terminal device control. Other human interfaces currently in development translate brain signals into prosthetic motor movements that allow users to control devices for greater precision and accuracy [44,45].

Amazon LLC and HERL have developed an alternative wearable device for individuals with SCI and MSK disorders who aim to return to work [46]. These wearable robots are seated mobile platforms designed to pick and place packages in fulfillment centers. Users are required to have the ability to move independently to the device and possess good upper extremity and trunk balance functions. These wearable robots may enhance function within the workspace, enabling users to perform tasks similarly to their able-bodied counterparts.

## 4. Discussion

### 4.1. Health Benefits

Research has shown a reduction in the MSK loads of critical body parts (e.g., back and shoulders) when using exoskeletons during selected static and dynamic tasks [47]. Back support exoskeletons can reduce hip and spinal muscle effort in forward-bending tasks [48], while upper limb exoskeletons can reduce shoulder muscle effort in arm-lifting tasks and prevent the occurrence of shoulder tendinopathies [49–51]. The utilization of exoskeletons is anticipated to alleviate the burden on soldiers’ backs and shoulders, possibly diminishing the likelihood of MSK injuries [47–51]. Improved ergonomics, which exoskeletons may promote, can be particularly beneficial in occupational settings where workers are prone to MSK disorders due to poor posture or repetitive motions.

Likewise, wearable robots may help by offloading some of the mechanical stress during repetitive tasks, reducing the likelihood of overuse injuries and health conditions such as arthritis. Some exoskeletons can be programmed to encourage proper movement patterns, potentially further minimizing the risk of joint-related conditions. Research findings indicate that employing an Aerial Porter Exoskeleton could lower the occurrence of MSK injuries among soldiers by enabling them to carry out tasks with reduced physical effort (e.g., lower heart rate, decreased oxygen consumption, and a decreased perception of exertion) [47,48,52]. This may not only reduce the risk of immediate injuries, but also may contribute to long-term MSK health.

Upper-extremity exoskeletons may offer benefits such as reducing spasticity and restoring dexterity through controlled reaching movements [53]. Lower-extremity exoskeletons may offer significant health benefits such as improving standing and walking function, gait patterns, walking speed, cardiovascular capacity, and the efficiency of oxygen consumption [54–61]. Early interventions using wearable assistive devices have shown improved rehabilitation outcomes [60]. Assistive mobility platforms enable people with impairments to perform functional tasks and provide ergonomic improvements for some users while maintaining work efficiency [46]. Wearable devices hold promise for alternative pathways for employment and career advancement.

### 4.2. Assessment Tools

Assessment tools for wearable robots are essential for evaluating their functionality, safety, and impact on users. Assessment tools help researchers, engineers, and healthcare professionals gather quantitative and qualitative data to make informed decisions about the functionality and optimal implementation of wearable robots. Functionality encompasses aspects such as usability, user acceptance, task performance, and comfort. Subjective parameters, including perceived exertion and user satisfaction, are typically measured using questionnaires, surveys, and semi-structured interviews. Muscle activity is often monitored using electromyography (EMG), while metabolic effects are often assessed through heart rate measurements, spirometry, and impedance cardiography [62]. Simulation models can be leveraged to investigate deeper muscle groups and compute joint loads [63]. The impact of wearable robots on human kinematics may be evaluated through motion capture systems using cameras and sensor tracking systems [30,64]. Force plates and tools to measure ground reaction forces and moments can assess the impact of wearable robots on gait and balance, and can be combined with EMG data to estimate muscle engagement and fatigue. Heart rate and physiological monitors can be used to assess cardiovascular response to the use of wearable robots, particularly relevant in applications involving physical exertion. Oxygen consumption (VO2) measurement may be used to quantify the metabolic cost of wearing an exoskeleton during different activities, providing insights into energy efficiency. Energy expenditure monitoring measures calories burned during activities with and without the wearable robot. Upper extremity function can be measured using standard hand function tests (e.g., nine-hole, box and block, Jebsen-Taylor Hand Function Test) [65]. Lower extremity function and mobility can be measured using static and dynamic balance tests (e.g., Time Up and Go test [66], Berg Balance Scale, and Functional Reach Test) [67] and gait tests (e.g., gait pattern and cycle tests, and gait analysis) [68]. Accuracy in task execution can assess the precision and effectiveness of wearable robot assistance during various activities.

Field studies and laboratory studies may be used for analyzing individual movements [30,64]. An innovative approach, such as the Exoworkathlon^®^, transfers real-world use cases into standardized laboratory settings with task-specific expert groups [69]. This approach enables an evaluation of exoskeleton effects, encompassing both subjective assessments through questionnaires and objective measurements through biomechanical measures. Safety checklists are used as systematic assessments of features and potential risks associated with wearable robot use. Assessment tools, used in combination, may provide a comprehensive understanding of the impact and effectiveness of wearable robots in various contexts, from rehabilitation to military applications.

### 4.3. Challenges and Areas of Opportunities

Wearable robots offer promising solutions for injury prevention and rehabilitation in military and Veteran populations for various fields in industries, healthcare, and the home/community. However, several challenges remain to be addressed for effective deployment and utilization. This study created some recommendations and considerations for exploring and adopting wearable robotic technologies, as follows:

#### Weight and Power consumption:

There is a gap between the need and ability of devices to be lightweight and to provide sufficient strength support during dynamic military tasks under high loads. Devices need to be able to produce a specific power between 50 and 300 W/kg, which is hard to achieve with present drive solutions like electric, pneumatic, or hydraulic drives. Long battery life and easily replaceable batteries are crucial for uninterrupted operation. Three paths may potentially minimize this challenge: (1) slower motion requirements can enable a lighter drive and a wearable robot, possibly practical for rehabilitation; (2) minimize assistance to only during specific targeted movements; and (3) control of passive springs, such as servo drives, may lead to active adaptive passive solutions which are lighter than active systems but more flexible than current passive wearable robots.

#### Flexibility:

Wearable robots need the ability to assist during specific activities. However, they must not hinder the user during other activities, especially in military applications [70]. Military tasks range from long-distance marches to complex maneuvers. Designing wearable robots that can adapt to the wide array of activities performed by military personnel poses a significant challenge.

#### Unknown long-term effects of wearable robots on users:

There is a paucity of long-term studies that aim to understand the effect of systems on reduced or increased load on the targeted joint complex and the neighboring MSK regions [51]. There is a need for further longitudinal studies on the entire postural chain during tasks when wearing an exoskeleton and the impact on static and dynamic posture. Effective use of wearable robots requires proper training, and soldiers will need time to integrate these new technologies. There needs to be an investigation into the impact on the user’s physical health, including potential strain on joints and muscles, as well as the physiological aspects of wearable robots [71,72]. The load on other body parts can increase depending on a task and wearable robot. Theurel and Desbrosess warned in 2018 [51] that passive wearable robots may lead to counterproductive antagonist muscle compensations and/or spinal imbalance as unintended effects.

#### Seamless integration with existing gear:

Soldiers must often carry heavy loads, and adding additional weight with wearable robots could exacerbate fatigue and limit mobility. Military operations often take place in diverse and challenging environments (e.g., desert, jungle, and extreme temperatures), requiring wearable robots to be robust and weather resistant. Wearable robots are not a one-stop solution for assistance, but a possible platform for different applications.

#### Independent use:

Recent advancements in wearable robots may allow for minimal assistance or independent donning and doffing, but they still pose challenges for people with disabilities. In addition, users may face challenges in independently recovering after a fall. If there is an unexpected power outage, finding a solution can be a hurdle without assistance. In such situations, individuals with disabilities may need assistance from a third party.

#### Training programs and evaluation:

It is necessary to implement comprehensive training programs to ensure that users can adapt and use the wearable robot effectively [73]. Increasing user comfort and confidence, for example through the development of standardized EAW training programs, is a critical factor for their successful adoption. These programs will need to be evaluated and validated for specific objectives (e.g., health benefits, cost–benefit).

#### Size, function, and customization:

While this analysis focused on the use of wearable robots for primary and secondary injury prevention; it is important to investigate and determine specific tasks or functions and target populations. For instance, people with disabilities include a wide range of etiologies that make it challenging to create one-size-fits-all upper- and lower-extremity wearable robots. Design requirements should be established to guide the customization of wearable robots according to user ergonomic needs.

#### Human–machine interaction:

Active wearable robots and users must work together to facilitate a ‘symbiotic’ interaction. The design of control algorithms for wearable robots needs to account for the response of the user to the forces generated by actuators [74]. In several applications, actuators need to react faster than physiological human responses for seamless and effective interaction [75]. Integrating biosensors in the design of wearable robots (e.g., to monitor the user’s biomechanics or enable EMG-control of actuators) can foster improvements in the ‘symbiotic’ human–machine interaction [76].

#### Affordability:

Wearable robots must become more affordable to be used in homes and communities, as they are still financially inaccessible to many individuals. It is crucial to analyze factors such as the initial cost, maintenance costs, and potential productivity or health benefits, making more wearable robots eligible for insurance coverage.

### 4.4. Study Limitation

Due to the short time to perform the modified Delphi consensus method in presence with the panelists and the steering group, the results were limited to qualitative findings. While results showed design considerations in wearable robots from experts in the field, future studies with a similar approach should consider supporting these findings with qualitative data.

## 5. Conclusions

In conclusion, this study aimed to define wearable robots for military rehabilitation and MSK injury prevention by capturing expert opinions. Through a modified Delphi process and thematic analyses, consensus was reached on the definition, categories, and applications of wearable robots. The study identified three main applications of wearable robots in military and rehabilitation settings, as follows: primary and secondary MSK injury prevention, enhancement of military activities and tasks, and functional rehabilitation and social reintegration. The classification of wearable robots provided insights into their structural and functional aspects. Furthermore, this study highlights the current state of technology, design requirements, and challenges associated with wearable robots. This research lays the foundation for future efforts aimed at advancing wearable robots for military and rehabilitation purposes, with the ultimate goal of improving the health and well-being of service members, veterans, and individuals with MSK injuries.

## Figures and Tables

**Table 1.** Classification of exoskeletons modified from La Tejera (2021) [22] and Looze (2016) [23].

Dimension			Specification			
Body Part	Full Body	Upper Body	Lower Body	Specific Segment	Specific Joint	Other
Structure	Rigid			Soft		
Action	Active		Semi-Active		Passive	
Powered Technology	Electric Actuator	Hydraulic Actuator	Pneumatic Actuator	Hybrid	Mechanical Systems	Others
Purpose	Rehabilitation			Assistance		
Application Area	Military	Healthcare	Research	Industrial	Civilian	Other Field
Intended Working Method	Static		Dynamic		Static and Dynamic	
Desired Application	Supporting Movement		Supporting Posture		Correcting Posture	

## Data Availability

The data presented in this study are available on request from the corresponding author.
